# Epidemiology and preventability of hospital-onset bacteremia and fungemia in 2 hospitals in India

**DOI:** 10.1017/ice.2023.170

**Published:** 2024-02

**Authors:** Sumanth Gandra, Sanjeev K. Singh, Murali Chakravarthy, Merlin Moni, Pruthu Dhekane, Zubair Mohamed, Fathima Shameen, Anil K. Vasudevan, Priyadarshini Senthil, Tejaswini Saravanan, Anu George, Dorothy Sinclair, Dustin Stwalley, Jacaranda van Rheenen, Matthew Westercamp, Rachel M. Smith, Surbhi Leekha, David K. Warren

**Affiliations:** 1 Division of Infectious Diseases, Department of Internal Medicine, Washington University School of Medicine in St. Louis, Missouri, United States; 2 Amrita Institute of Medical Sciences, Kochi, Kerala, India; 3 Fortis Hospital, Bengaluru, Karnataka, India; 4 Centers for Disease Control and Prevention, Atlanta, Georgia, United States; 5 Division of Infectious Diseases, Department of Internal Medicine, University of Maryland Medical School, Baltimore, Maryland, United States

## Abstract

**Objective:**

Studies evaluating the incidence, source, and preventability of hospital-onset bacteremia and fungemia (HOB), defined as any positive blood culture obtained after 3 calendar days of hospital admission, are lacking in low- and middle-income countries (LMICs).

**Design, setting, and participants:**

All consecutive blood cultures performed for 6 months during 2020–2021 in 2 hospitals in India were reviewed to assess HOB and National Healthcare Safety Network (NHSN) reportable central-line–associated bloodstream infection (CLABSI) events. Medical records of a convenience sample of 300 consecutive HOB events were retrospectively reviewed to determine source and preventability. Univariate and multivariable logistic regression analyses were performed to identify factors associated with HOB preventability.

**Results:**

Among 6,733 blood cultures obtained from 3,558 hospitalized patients, there were 409 and 59 unique HOB and NHSN-reportable CLABSI events, respectively. CLABSIs accounted for 59 (14%) of 409 HOB events. There was a moderate but non-significant correlation (r = 0.51; *P* = .070) between HOB and CLABSI rates. Among 300 reviewed HOB cases, CLABSIs were identified as source in only 38 (13%). Although 157 (52%) of all 300 HOB cases were potentially preventable, CLABSIs accounted for only 22 (14%) of these 157 preventable HOB events. In multivariable analysis, neutropenia, and sepsis as an indication for blood culture were associated with decreased odds of HOB preventability, whereas hospital stay ≥7 days and presence of a urinary catheter were associated with increased likelihood of preventability.

**Conclusions:**

HOB may have utility as a healthcare-associated infection metric in LMIC settings because it captures preventable bloodstream infections beyond NHSN-reportable CLABSIs.

The Centers for Disease Control and Prevention (CDC) National Healthcare Safety Network (NHSN) central-line–associated bloodstream infection (CLABSI) metric is a widely accepted quality measure for hospital infection-prevention activities. However, CLABSI surveillance can be resource intensive, even in the United States, and can suffer from subjectivity.^1^ In low- and middle-income countries (LMICs), where both human and monetary resources are limited, a more objective, simple, and easily automated healthcare-associated infection (HAI) surveillance metric is needed. One such metric is hospital-onset bacteremia and fungemia (HOB), which includes not only CLABSI but also secondary bloodstream infections due to any other healthcare-acquired infections, such as urinary tract or respiratory tract infections. Moreover, HOB can potentially be collected from microbiology data alone and can provide a wider view of HAIs beyond NHSN-reportable CLABSIs, many of which may be preventable and targets for infection prevention activities. A preliminary US study indicated that ∼50% of all HOB events, excluding contaminants, are potentially preventable.^2^ However, the incidence, causes, and overall preventability of HOB is unknown in LMICs. The objectives of the study were (1) to assess the incidence of HOB and NHSN-reportable CLABSI, (2) to assess the sources and preventability of HOB events, and (3) to determine the feasibility of conducting laboratory-based HOB surveillance in 2 hospitals in India.

## Methods

### Setting, HOB definition, and microbiology methods

This study was conducted in 2 tertiary-care hospitals (hospitals A and B) in South India. HOB was defined as any growth of microorganism, including potential contaminants, from a blood culture obtained at least 3 calendar days after hospital admission, with the admission date considered as day 1.^2^ All consecutive blood cultures processed for 6 months in the microbiology laboratory were captured prospectively from the laboratory registry. In hospital A, all consecutive blood cultures performed between August 16, 2020, and February 15, 2021, were analyzed; in hospital B, all blood cultures performed between January 1, 2021, and June 30, 2021, were analyzed. A convenience sample of 300 consecutive HOB cases (200 and 100 consecutive HOB cases in hospitals A and B, respectively) were examined for source of infection and preventability by retrospective medical chart review. This study was approved by the Human Research Protection Office at Washington University School of Medicine (ID no. 202001017), the 2 study hospitals’ ethics committees (ID nos. 2020-002 and IEC/011/2020), and the Indian Health Ministry’s Screening Committee.

Hospital A is a 1,250-bed, private, medical college and tertiary-care hospital, whereas hospital B is a 300-bed, private, tertiary-care hospital. Both hospitals have onsite diagnostic microbiology laboratories that are accredited by the Indian National Accreditation Board for Testing & Calibration Laboratories (Table 1). The microbiology laboratories at both hospitals are equipped with BacT/ALERT (bioMérieux, Marcy-l’Étoile, France) automated blood-culture systems for processing blood cultures and VITEK2 (bioMérieux, Marcy-l’Étoile, France) automated platforms to perform organism identification as well as antimicrobial susceptibility testing (AST), with regular quality-control processes in place. The 2 study hospitals conduct device-associated HAI and surgical-site infection (SSI) surveillance based on the CDC NHSN criteria.^3^

**Table 1.** Characteristics of the 2 Study Hospitals in India

Hospital Characteristics	Hospital A	Hospital B
Type of hospital	Private, tertiary-care teaching	Private, tertiary care
Total beds	1,250	300
Medical ICU beds	124	24
Surgical ICU beds	124	24
Oncology beds	36	18
Neurology ICU beds	14	12
Pediatric ICU beds	8	6
Neonatal ICU beds	22	12
Liver, kidney, and bone-marrow transplant services	Available	Available
Trauma services	Available	Available
Electronic medical records	Partial	Partial
Indigent patient services available	No	No
No. of full-time infection prevention nurses	5	2
Device-associated HAI and SSI surveillance conducted using CDC-NHSN criteria	Yes	Yes
Mandatory HAI reporting to national accreditation body	Yes (monthly)	Yes (monthly)
Clinical microbiologist, full time	Yes	Yes
Blood-culture system (automated)	BacT/ALERT	BacT/ALERT
Organism identification and antimicrobial susceptibility platform	VITEK2	VITEK2
National Accreditation Board for Testing and Calibration Laboratories, India Accreditation	Yes	Yes

Note. ICU, intensive care unit; HAI, healthcare-associated infection; SSI, surgical-site infection; CDC, Centers for Disease Control and Prevention; NHSN, National Healthcare Safety Network.

### Data collection

The following data were collected for each blood culture: patient demographic data, coronavirus disease 2019 (COVID-19) status, hospital admission date, and date admitted to ward or ICU, specimen collection date and location (outpatient, emergency room, or inpatient), final result (growth or no growth), organism identification, and AST results. If a positive blood culture met the study HOB definition, then the following information was collected: the blood-culture source (ie, whether drawn from central line, peripheral vein stick, arterial line, or unknown) and whether it met NHSN-reportable CLABSI criteria, as determined by the individual hospital’s CLABSI surveillance program. Monthly patient days and central-line days were obtained from the hospital information system and infection prevention database, respectively. Duplicate positive blood cultures were defined as having at least 1 matching organism in blood culture within a 14-day period. If a patient had multiple positive cultures that met the HOB definition but with different organisms within a 14-day period, they were considered separate HOB events. Blood-culture contamination was defined as the isolation of 1 or more common commensal organisms listed on the CDC NHSN 2022 list in only a single blood culture in 1 set or 1 of a series of 2 or more blood cultures.

For the 300 selected HOB cases, a detailed data collection form was created to capture the following information: reason for admission, acute and chronic comorbid conditions, indication for blood culture, details of any surgical procedures performed 30 days prior to HOB, other invasive procedures performed in prior 14 days, devices present on the day or within 2 calendar days of the index positive blood culture, clinical findings and hospital course prior to the index HOB event, microbiological cultures from other specimens 7 days before and 7 days after the index positive blood culture, and antibiotic treatment. The source of each HOB was determined using clinical criteria based on clinician review and judgment.

### Framework development for the preventability of HOBs

A framework to determine the preventability of an HOB event was adapted from US studies^2,4^ by including medical conditions encountered in LMICs (Supplementary Table 1). Then, 10 subject-matter experts (Supplementary Table 2) evaluated the HOB preventability framework through an online survey and an in-person meeting that was held on November 20, 2019, at Washington University School of Medicine in St. Louis. In this framework, the preventability of HOB is conceptualized as a function of both patient intrinsic risk for developing bacteremia, and extrinsic hospital practices, including patient care and infection prevention. The preventability of each HOB was assessed on a 6-point Likert scale using a matrix which incorporates comparative risk of bacteremia due to underlying conditions on 1 axis and the likelihood of preventing the infection type under ideal conditions on the other axis.^2^ The preventability rating is based on an “ideal hospital” that practices “flawless infection control and patient care even in resource-limited settings.”^2^ The 6-point Likert-scale scoring was structured as follows: 1 (definitely preventable), 2 (probably preventable), 3 (more likely preventable than not), 4 (less likely preventable than not), 5 (probably not preventable), and 6 (definitely not preventable). HOB events rated 1–3 were considered potentially preventable whereas those rated 4–6 were considered not preventable.^2^ All data were entered into a REDCap database.

**Table 2.** Hospital-Onset Bacteremia and Fungemia (HOB) Characteristics in 2 Hospitals in India During 2020–2021

Variable	Total, No. (%)^a^	Hospital A, No. (%)^a^	Hospital B, No. (%)^a^	*P* Value
Hospital-wide PD	142,568	113,371	29,197	…
Blood culture rate per 1,000 PD	55.19	48.81	79.97	<.001
HOB events (unique)	409	301	108	…
Unique patients with HOB	372	275	97	…
HOB rate per 1,000 PD	2.87	2.65	3.7	.003
Time from admission to HOB event, median d (IQR)	15 (8–23)	15 (8–23)	15 (8.5–23)	.780
**Blood-culture source**				
Central line	151 (32)	111 (32)	40 (32)	.970
Peripheral vein	288 (62)	214 (62)	74 (60)	.610
Arterial line	14 (3)	5 (2)	9 (7)	.010
Unknown	15 (3)	14 (4)	1 (1)	.080
Medical ICU HOB rate per 1,000 PD	14.6	12.58	17.82	.110
Surgical ICU HOB rate per 1,000 PD	8.64	9.5	6.81	.360
NICU HOB rate per 1,000 PD	11.2	8.77	11.44	.720
Pediatric ICU HOB rate per 1,000 PD	14.59	17.43	0.00	NC
Wards HOB rate per 1,000 PD	1.82	1.71	2.32	.046
**Microbiology**				
Gram positive	163 (31)	108 (28)	55 (40)	.010
Coagulase-negative *Staphylococcus*	89 (17)	53 (14)	36 (26)	.001
*Enterococcus* spp	43 (8)	29 (8)	14 (10)	.350
*Streptococcus* spp	11 (2)	8 (2)	3 (2)	.950
*Staphylococcus aureus*	10 (2)	8 (2)	2 (1)	.640
Others	10 (2)	10 (3)	0 (0)	.060
Gram negative	312 (60)	236 (61)	76 (55)	.170
*Klebsiella* spp	128 (24)	98 (26)	30 (22)	.360
*Escherichia coli*	46 (9)	37 (10)	9 (7)	.260
*Acinetobacter* spp	46 (9)	39 (10)	7 (5)	.070
*Burkholderia* spp	18 (4)	10 (3)	8 (7)	.080
*Pseudomonas aeruginosa*	18 (4)	13 (3)	5 (4)	.900
Others	56 (11)	39 (10)	17 (12)	.490
Fungi	49 (9)	41 (10)	8 (7)	.090
*Candida* spp	34 (7)	29 (8)	5 (4)	.170
*Candida auris*	13 (3)	11 (3)	2 (1)	.370
Contamination rate among HOB per 1,000 PD	0.26	0.12	0.78	<.001

Note. PD, patient days; IQR, interquartile range; ICU, intensive care unit; NICU, neonatal ICU; NC, not calculable.

a Units unless otherwise specified.

Prior to data collection on HOB source and preventability, US investigators conducted online training sessions with the study teams at the 2 hospitals (Supplementary Box 1 online). To assess the feasibility of laboratory-based HOB surveillance and barriers faced during data collection, a qualitative group interview session was conducted with the study team separately at each hospital, using a semistructured interview approach that included open-ended questions (Supplementary Appendix 1 online).^5^

### Data analysis

*HOB and NHSN-reportable CLABSI incidence and characteristics.* Descriptive analyses were performed to examine the frequencies, rates, and organism distribution of HOB and CLABSI events after excluding duplicates using the criteria defined above. The χ^2^ or Fisher exact test was utilized for categorical variables and Mann–Whitney *U* tests were utilized for continuous variables. HOB rates and blood-culture contamination rates were calculated as the total number of events divided by total patient days, and CLABSI rates were calculated as the total number CLABSIs divided by total central-line days. Rates were compared using Poisson regression. Correlation between CLABSI and HOB rates in ICUs was assessed using the Spearman rank correlation. We only included ICUs for correlation because central-line utilization outside the ICU was minimal in these hospitals.

*Preventability of HOB.* For the 300 HOB cases, frequency distributions of sources of HOB and other clinical attributes of HOB including pathogen distribution, antimicrobial resistance proportion, and presence of invasive devices were calculated. The proportion of potentially preventable HOB cases was also determined. To identify demographic and clinical factors associated with HOB preventability, univariate and multivariable analyses were performed. Univariable analyses were performed using the χ^2^ or Fisher exact test and variables with *P* < .20 were considered in backward elimination selection for a multivariable logistic regression model. However, we forced “hospital” variable in the multivariable analysis because there were some inherent differences between the 2 facilities (Table 1). *P* < .05 was considered statistically significant. All data analyses were performed in SAS version 9.4 software (SAS Institute, Cary, NC).

## Results

### Incidence of HOB and NHSN-reportable CLABSIs

**Figure 1.**
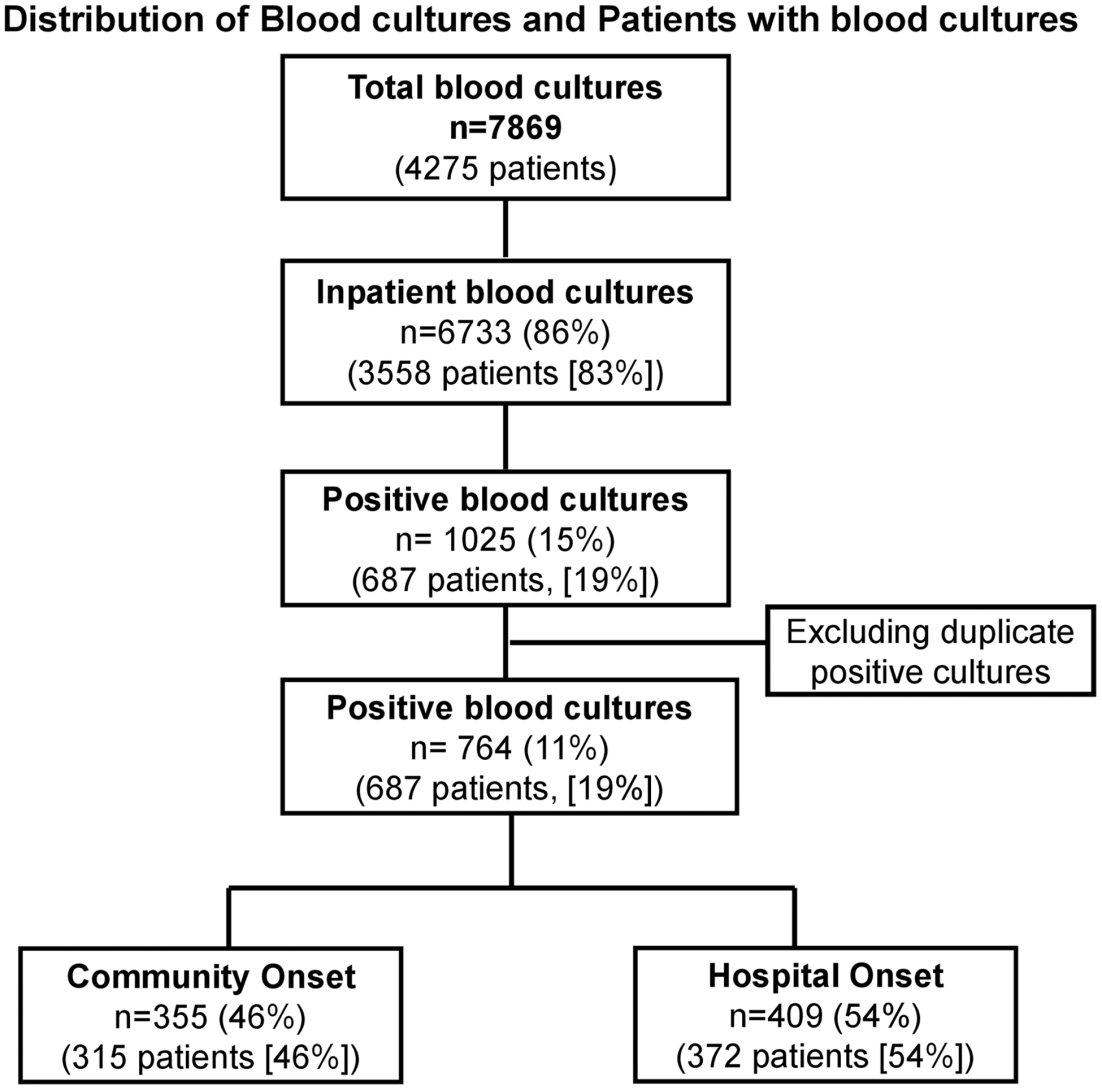
Distribution of blood cultures, patients with blood cultures and number of hospital-onset bacteremia and fungemia (HOB) cases at the 2 study hospitals in India during 2020–2021.

Overall, 6,733 blood cultures were obtained from 3,558 hospitalized patients from the 2 study hospitals (Fig. 1). After excluding duplicate positive cultures among 6,733 blood cultures, 764 (11%) were positive, with 409 unique HOB events in 372 patients. Compared to hospital A, hospital B had significantly higher rates of HOB (3.7 vs 2.65 of 1,000 patient days; *P* = .003), and blood-culture contamination (0.78 vs 0.12 per 1,000 patient days; *P* < .001) (Table 2). No significant difference in HOB rate was observed among ICUs in 2 hospitals, but the HOB rate in wards was significantly higher in hospital B (2.32 vs 1.71 per 1,000 patient days; *P* = .046). In both hospitals, gram-negative organisms were more frequently observed (60%) compared to gram-positive and fungal organisms in HOB events.

There were 59 NHSN-reportable CLABSI events in both hospitals, and they accounted for 59 (14%) of 409 of HOB cases. The CLABSI rate was significantly higher in hospital A than hospital B (6.37 vs 1.34 per 1,000 central-line days; *P* < .001) (Table 3). No significant difference in CLABSI rates was observed between the medical and surgical ICUs in the 2 hospitals. CLABSI rates were higher in pediatric and neonatal ICUs in hospital A compared to hospital B, where no CLABSIs were reported in this group. Like HOB, gram-negative organisms were more frequently isolated (70%) among CLABSIs in both hospitals, compared to gram-positive and fungal organisms. There was a moderate but nonsignificant correlation (r = 0.51; *P* = .07) between HOB and CLABSI rates among ICUs for the 2 hospitals.

**Table 3.** National Healthcare Safety Network (NHSN) Central-Line–Associated Bloodstream Infection (CLABSI) Characteristics in 2 Hospitals in India during 2020–2021

Variable	Total, No. (%)^a^	Hospital A, No. (%)^a^	Hospital B, No. (%)^a^	*P* Value
No. of CLABSIs	59	52	7	…
Total central-line days	13400	8164	5236	…
CLABSI rate per 1,000 central-line days	4.4	6.37	1.34	<.001
Medical ICU CLABSI rate	5.48	6.20	4.44	.590
Surgical ICU CLABSI rate	3.78	5.51	0.91	.090
Neonatal ICU CLABSI rate	13.93	14.96	0.00	NC
Pediatric ICU CLABSI rate	12.57	14.45	0.00	NC
**Microbiology**				
Gram positive	14 (16)	11 (15)	3 (25)	.350
Coagulase-negative *Staphylococcus*	3 (3)	3 (4)	0 (0)	.480
*Enterococcus* spp	7 (8)	5 (7)	2 (17)	.230
*Streptococcus* spp	1 (1)	0 (0)	1 (8)	.010
*Staphylococcus aureus*	2 (2)	2 (3)	0 (0)	.570
Others	1 (1)	1 (1)	0 (0)	.690
Gram negative	65 (74)	56 (74)	9 (75)	.920
*Klebsiella* spp	27 (31)	23 (30)	4 (33)	.830
*Escherichia coli*	2 (2)	1 (1)	1 (8)	.130
*Acinetobacter* spp	13 (15)	13 (17)	0 (0)	.120
*Burkholderia* spp	3 (3)	3 (4)	0 (0)	.480
*Pseudomonas aeruginosa*	4 (4)	3 (4)	1 (8)	.500
Others	16 (18)	13 (17)	3 (25)	.510
Fungi	9 (10)	9 (12)	0 (0)	.210
*Candida* spp	6 (7)	6 (8)	0 (0)	.310
*Candida auris*	3 (3)	3 (4)	0 (0)	.480

Note. NC, not calculable; ICU, intensive care unit.

a Units unless otherwise specified.

### Source and preventability of HOB

Among the 300 HOB consecutive cases that underwent detailed chart review, half of the patients underwent surgery or invasive procedure. Central venous catheters were present in 234 (78%) of 300 patients, and urinary catheters were present in 174 (58%) of these 300 patients. COVID-19 was diagnosed in 61 (20%) of 300 patients. Also, 177 (59%) of 300 patients were in ICUs at the time of the HOB event, and 57 (19%) 300 patients were neutropenic. Central-line infection (based on clinical adjudication) was the most common source of HOB, accounting for 79 (26%) of 300 HOB cases (Table 4). NHSN-reportable CLABSI accounted for 38 (13%) of 300 HOB cases. Device-associated infections (central lines, urinary catheter, and mechanical ventilator) as source of HOB accounted for 96 (32%) of 300 HOB cases.

**Table 4.** Patient Demographic, Clinical Characteristics and Causes of 300 Hospital-Onset Bacteremia and Fungemia (HOB) Cases in 2 Study Hospitals in India during 2020–2021

Variable	Total (N = 300), No. (%)^a^	Hospital A (N = 200), No. (%)^a^	Hospital B (N = 100), No. (%)^a^	*P* Value
**Demographics**				
Sex, male	184 (61)	131 (66)	53 (53)	.040
Age, median y (IQR)	52 (24–66)	51.5 (6.5–62)	55 (37–69)	.002
Time from admission to HOB event, median d (IQR)	14 (9–21)	14 (9–21)	14 (8–21)	.900
**Patient location**				
Intensive care unit (ICU)	177 (59)	127 (64)	59 (50)	.030
All adult ICU	134 (45)	86 (43)	48 (48)	.410
Neonatal ICU	29 (10)	27 (14)	2 (2)	.002
Pediatric ICU	14 (5)	14 (7)	0 (0)	.007
Ward	123 (41)	73 (37)	50 (50)	.030
**Comorbidities**				
Cardiorespiratory arrest or CPR prior to index culture	26 (9)	12 (6)	14 (14)	.020
Burns	15 (5)	8 (4)	7 (7)	.260
Dialysis	29 (10)	22 (11)	7 (7)	.270
Decubitus ulcer	26 (9)	17 (9)	9 (9)	.880
Neutropenia	57 (19)	34 (17)	23 (23)	.210
Shock	34 (11)	14 (7)	20 (20)	.001
Total parenteral nutrition	26 (9)	22 (11)	4 (4)	.040
Active malignancy with chemotherapy	19 (6)	10 (5)	9 (9)	.180
Acute respiratory distress	63 (21)	19 (10)	44 (44)	<.001
Renal failure	16 (5)	11 (6)	5 (5)	.860
Pneumonia	53 (18)	21 (11)	32 (32)	<.001
Chronic kidney disease	23 (8)	16 (8)	7 (7)	.760
Coronary artery disease	26 (9)	16 (8)	10 (10)	.560
Diabetes mellitus	44 (15)	19 (10)	25 (25)	<.001
Cirrhosis of liver	35 (12)	30 (15)	5 (5)	.010
Hematologic malignancy	22 (7)	18 (9)	4 (4)	.120
Solid tumor	30 (10)	22 (11)	8 (8)	.410
COVID-19 diagnosis	61 (20)	35 (18)	26 (26)	.080
**Indwelling devices and procedures**				
Central venous catheter (any)	234 (78)	147 (74)	87 (87)	.008
Temporary central line	188 (63)	120 (60)	68 (68)	.180
Peripherally inserted central catheter	38 (13)	24 (12)	14 (14)	.620
Port or tunneled catheter	2 (1)	0 (0)	2 (2)	.110
Dialysis catheter	23 (8)	13 (7)	10 (10)	.280
Midline catheter	1 (0)	0 (0)	1 (1)	.330
Urinary catheter	173 (58)	97 (49)	76 (76)	<.001
Mechanical ventilator support	103 (34)	57 (29)	46 (46)	.003
Arterial line	137 (46)	81 (41)	56 (56)	.010
Other indwelling device	216 (72)	172 (86)	44 (44)	<.001
No indwelling device	3 (1)	2 (1)	1 (1)	1.000
Surgery or other invasive procedure	150 (50)	114 (57)	36 (36)	<.001
In-hospital mortality	93 (31)	61 (31)	32 (32)	.790
**Microbiology**				
Gram positive	93 (31)	54 (24)	39 (38)	.010
Coagulase-negative *Staphylococcus*	51 (17)	25 (11)	26 (25)	.001
*Enterococcus* spp	23 (8)	15 (7)	8 (8)	.730
*Streptococcus* spp	5 (2)	3 (1)	2 (2)	.650
*Staphylococcus aureus*	4 (1)	3 (1)	1 (1)	1.000
Gram negative	183 (61)	131 (59)	52 (51)	.160
*Klebsiella* spp	75 (25)	56 (25)	19 (19)	.180
*Escherichia coli*	30 (10)	22 (10)	8 (8)	.540
*Acinetobacter* spp	27 (9)	21 (10)	6 (6)	.270
*Burkholderia* spp	13 (4)	7 (3)	6 (6)	.360
*Pseudomonas aeruginosa*	9 (3)	8 (4)	1 (1)	.280
*Enterobacter* spp	9 (3)	5 (2)	4 (4)	.470
*Stenotrophomonas maltophilia*	7 (2)	3 (1)	4 (4)	.210
Fungi	37 (12)	30 (14)	7 (7)	.080
*Candida* spp	35 (12)	28 (13)	7 (7)	.120
*Candida auris*	10 (3)	8 (4)	2 (2)	.420
**Source of bacteremia or fungemia**				
Endovascular	88 (29)	62 (31)	26 (26)	.370
Central line	79 (26)	54 (27)	25 (25)	.710
NHSN-reportable CLABSI	38 (13)	32 (16)	6 (6)	.010
Arterial line	7 (2)	6 (3)	1 (1)	.430
Peripheral IV catheter	2 (1)	2 (1)	0 (0)	.550
Unknown	58 (19)	36 (18)	22 (22)	.410
Skin contamination	55 (18)	30 (15)	25 (25)	.030
Mucosal barrier injury	30 (10)	17 (9)	13 (13)	.220
Gastrointestinal tract	24 (8)	19 (10)	5 (5)	.180
Respiratory tract	23 (8)	18 (9)	5 (5)	.220
Mechanical ventilator	8 (3)	5 (3)	3(3)	1.000
Urinary tract	14 (5)	11 (6)	3 (3)	.400
Urinary catheter	9 (3)	7 (4)	2 (2)	.720
Skin and soft tissue	8 (3)	7 (4)	1 (1)	.280

Note. IQR, interquartile range; ICU, intensive care unit; PICC, peripherally inserted central catheter; NHSN, National Healthcare Safety Network; CLABSI, central-line–associated bloodstream infection.

a Units unless otherwise specified.

**Figure 2.**
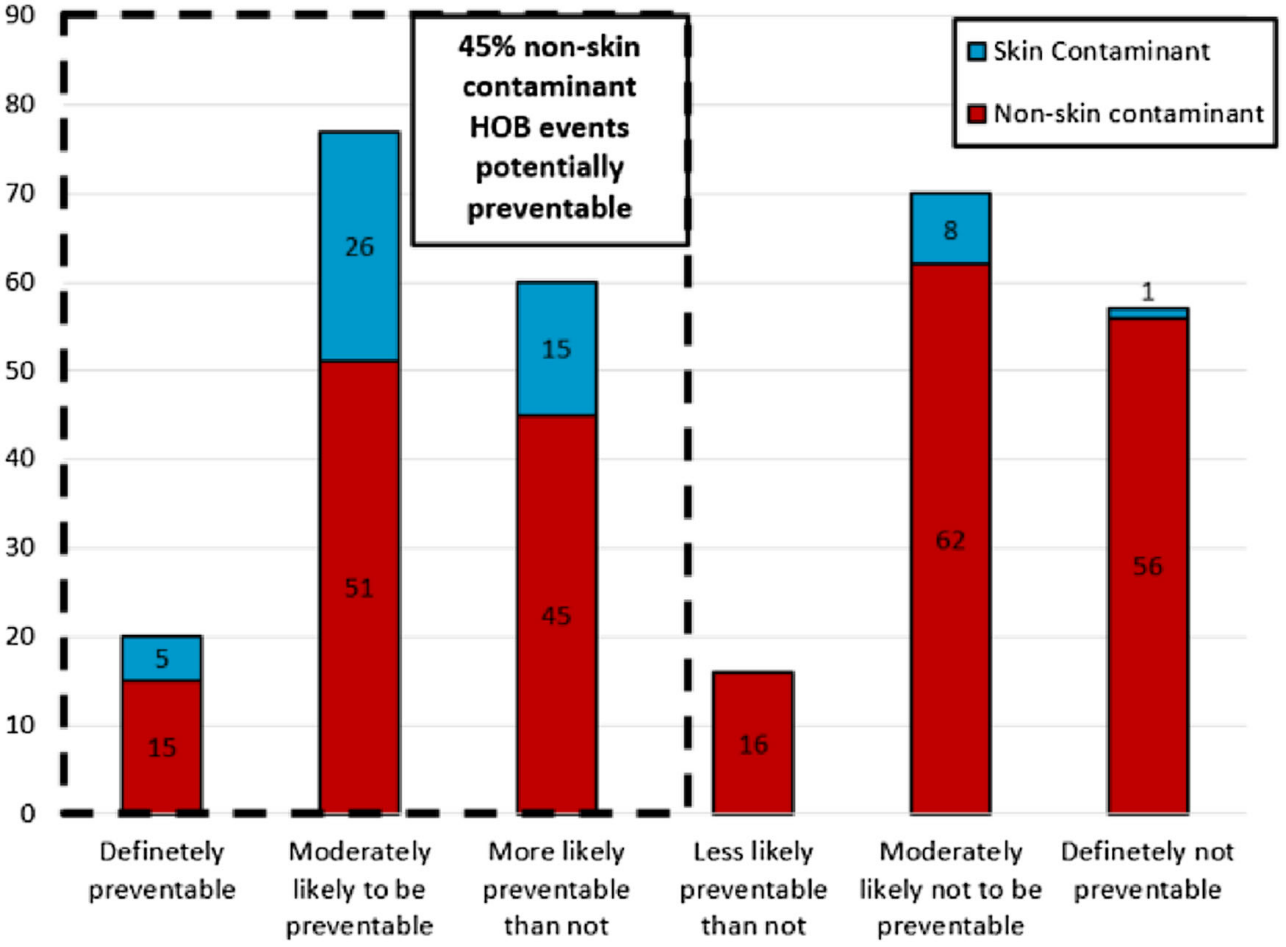
Preventability rating of hospital-onset bacteremia and fungemia (HOB) cases in 2 hospitals in India during 2020–2021 (n = 300).

Overall, 157 (52%) of 300 HOB cases, and 45% of HOB cases not attributable to skin contaminants, were rated as potentially preventable (Fig. 2). Central lines were the source for 76 (48%) of 157 preventable HOB cases in clinical adjudication, and NHSN-reportable CLABSI accounted for 22 (14%) of 157 preventable HOB cases. Several variables were significantly associated with preventability in univariate analysis (Table 5). The highest magnitudes of association were observed for a central-line source (OR, 43.8; 95% CI, 13.6–221.6) and skin contaminants (odds ratio [OR], 6.2; 95% confidence interval [CI], 2.9–13.2). NHSN-reportable CLABSI was not associated with preventable HOB (OR, 1.3; 95% CI, 0.7–2.6). In the multivariable analysis, neutropenia (OR, 0.2; 95% CI, 0.1–0.4) and sepsis as an indication for blood culture (OR, 0.38; 95% CI, 0.2–0.7) were associated with decreased odds of HOB preventability, whereas hospital stay ≥7 days (OR, 3.3; 95% CI, 1.7–6.7) and the presence of urinary catheter (OR, 1.8; 95% CI, 1.0–3.1) were associated with increased likelihood of preventability (Table 5). Central lines and skin contaminants as source of HOB, although significantly associated with HOB preventability in univariable analysis, were not included in the multivariable analysis due to small numbers in nonpreventable category.

**Table 5.** Univariable and Multivariable Analysis of Factors Associated With Preventability of Hospital-Onset Bacteremia and Fungemia (HOB) in 2 Hospitals in India During 2020–2021

Variable	HOB Preventable (N = 157), No. (%)^a^	HOB Nonpreventable (N = 143), No. (%)^a^	Univariable Analysis, Odds Ratio [95% CI]	Multivariable Analysis Odds Ratio [95% CI]
**Hospital** Hospital A Hospital B	103 (52) 54 (54)	97 (49) 46 (46)	Reference 1.1 [0.7–1.8]	1.2 [0.7–2.1]
Sex, female	55 (35)	61 (43)	0.7 [0.5–1.2]	NS
**Age**				
≥60 y	68 (43)	39 (27)	2.0 [1.3–3.3]	NS
Pediatric (<19 y)	32 (20)	37 (26)	0.7 [0.4–1.3]	NS
Adults (>18 y)	125 (80)	106 (74)	1.4 [0.8–2.3]	…
Hospital stay ≥ 7 d	141 (90)	112 (78)	2.4 [1.3–4.7]	3.3 [1.7–6.7]
**Location**				
Intensive care unit	94 (60)	83 (58)	1.1 [0.7–1.7]	…
Ward	62 (40)	55 (39)	1.0 [0.7–1.7]	…
Immunosuppressive therapy	36 (23)	60 (42)	0.4 [0.3–0.7]	NS
Cardiorespiratory arrest	8 (5)	18 (13)	0.4 [0.2–0.9]	NS
Burns	11 (7)	4 (3)	2.6 [0.8– 1.5]	NS
Dialysis	16 (10)	13 (9)	1.3 [0.5–2.5]	…
Decubitus ulcer	16 (10)	10 (7)	1.5 [0.7–3.4]	…
Neutropenia	12 (8)	45 (32)	0.2 [0.1–0.4]	0.2 [0.1–0.5]
Shock	13 (8)	21 (15)	0.5 [0.3–1.1]	NS
Total parenteral nutrition	13 (8)	13 (9)	0.9 [0.4–2.0]	…
Active chemotherapy	4 (3)	15 (11)	0.2 [0.1–0.7]	NI
Acute respiratory distress	35 (22)	28 (20)	1.2 [0.7–2.1]	…
Renal failure	7 (5)	9 (6)	0.7 [0.3–1.9]	…
Pneumonia, ICU	36 (23)	16 (11)	2.5 [0.3–4.6]	NS
Chronic kidney disease	13 (8)	10 (7)	1.2 [0.5–2.8]	…
Coronary artery disease	18 (12)	8 (6)	2.2 [0.9–5.1]	NS
Diabetes	22 (14)	22 (15)	0.9 [0.5–1.7]	…
Cirrhosis	16 (10)	19 (13)	0.7 [0.4–1.5]	…
Hematologic malignancy	6 (4)	16 (11)	0.3 [0.1– 0.8]	NI
Solid tumor	16 (10)	14 (10)	1.1 [0.5– 2.2]	…
Surgery or procedure	79 (50)	71 (50)	1.0 [0.7–1.6]	…
**Device**				
Central line (any)	128 (82)	106 (74)	2.0 [1.3–3.2]	NS
Urinary catheter	103 (67)	70 (49)	1.5 [0.9–2.7]	1.8 [1.0–3.1]
Arterial line	83 (53)	54 (38)	1.9 [1.2–2.9]	NS
Mechanical ventilator	57 (36)	46 (32)	1.2 [0.7–1.9]	…
PEG/PEG–J tube	8 (5)	8 (6)	0.9 [0.3–2.5]	…
Tracheostomy	24 (15)	11 (8)	2.2 [1.0–4.6]	NS
Drain	10 (6)	8 (6)	1.2 [0.4–3.0]	…
Temporary CVC	114 (73)	74 (52)	2.5 [1.5–4.0]	NS
Hemodialysis catheter	17 (11)	6 (4)	2.8 [1.1–7.2]	NS
PICC	11 (7)	27 (19)	0.3 [0.2–0.7]	NS
**Blood culture indication**				
Fever	127 (80)	107 (75)	1.4 [0.8–2.5]	…
Sepsis	62 (40)	79 (55)	0.5 [0.3–0.8]	0.4 [0.2–0.6]
COVID–19 diagnosis	44 (28)	17 (12)	2.9 [1.6–5.3]	NS
**Source**				
Skin and soft tissue	3 (2)	5 (4)	0.5 [0.1–2.8]	–
Gastrointestinal	2 (1)	22 (15)	0.1 [0.0–0.30]	NI
Endovascular	85 (54)	3 (2)	55.1 [17.1–278.4]	NI
Central line	76 (48)	3 (2)	43.78 [13.6–221.6]	NI
NHSN reportable CLABSI	22 (14)	16 (11)	1.3 [0.7–2.6]	…
Urinary	9 (6)	5 (4)		NI
Urinary catheter	9 (5)	0 (0)	…	NI
Respiratory	11 (7)	12 (8)	0.8 [0.4–1.9]	…
Mechanical ventilator	8 (5)	3 (2)	2.5 [0.6–14.9]	NI
Mucosal barrier injury	1 (1)	29 (20)	0.03 [0.0– 0.2]	NI
Contaminant	46 (29)	9 (6)	6.2 [2.9–13.2]	NI
None identified	0 (0)	58 (41)	…	NI

Note. NS, not significant; NI, not included due to numbers <10; ICU, intensive care unit; PEG/PEG-J, percutaneous endoscopic gastrostomy/percutaneous endoscopic gastrojejunostomy; CVC, central venous catheter; COVID-19, coronavirus disease 2019; PICC, peripherally inserted central catheter.

a Units unless otherwise specified.

### Feasibility of conducting HOB surveillance

In qualitative group interviews, research staff at both hospitals did not report any barriers on collecting data related to blood cultures and patient days. However, both hospitals staff indicated that in some cases collecting data to determine source and preventability of HOB was challenging because the documentation was handwritten and sometimes difficult to understand or was otherwise incomplete. Additionally, preventability and source of HOB could not always be clearly determined due to lack of accompanying diagnostic tests. On average for each HOB case, it took 20 minutes to determine the source and preventability. From a feasibility perspective, although both hospitals have laboratory information systems where blood-culture data can be accessed, date of admission is currently not included as a discrete field in the blood-culture requisition form. Both hospitals have capacity to link each blood culture to hospital information system to obtain the admission date, and an alternative adding admission date in blood culture requisition form and into laboratory information systems is feasible with minimal resources. Currently in the 2 hospitals, to implement NHSN-based CLABSI surveillance, it takes ∼3–4 hours per day of combined effort from all infection prevention nurses. Implementing HOB surveillance to determine the source and preventability of each HOB case will take >10 hours per day of combined effort from all infection prevention nurses.

## Discussion

Recent interest in HOB as a quality metric in the United States has increased^6–8^ because it can often be collected from microbiology data alone. Many LMICs have attempted to implement NHSN-based CLABSI surveillance with extremely constrained resources, and HOB holds great potential as an alternative. However, studies examining the epidemiology, preventability of HOB, and feasibility of implementing HOB surveillance in LMICs are lacking. Here, we present the results of our CLABSI surveillance investigation in 2 hospitals in India.

We observed that NHSN-reportable CLABSIs accounted for only 14% of all HOB events and that there was a moderate but nonsignificant correlation between NHSN-reportable CLABSI and HOB rates. Overall, HOB and CLABSI events identified similar organisms causing HAIs and gram-negative organisms predominated, with *Klebsiella* spp being most common. Conversely, in the United States, gram-positive organisms predominate as causes of CLABSI and HOB.^9,10^

We observed that central-line infections were the most common clinically adjudicated source of HOB, accounting for 26% of HOB cases, and NHSN-reportable CLABSI accounted for only 13% of HOB cases. Thus, NHSN-CLABSI surveillance may be missing as many as half of all bloodstream infections attributed to central-line infections in this setting. Furthermore, in this study, 52% of all HOB cases and 45% of non–skin-commensal HOB cases were judged to be preventable in an ideal setting when recommended infection prevention practices are followed. Central lines were considered the source of 48% of preventable HOB cases, in contrast to NHSN-reportable CLABSIs, which constituted only 14% of preventable HOB cases, respectively. In addition, we identified other preventable sources of HOB, such as peripheral venous catheters, arterial catheters and skin and soft-tissue infections, which are not captured in routine CLABSI surveillance. Thus, HOB could be a potential HAI metric because it captures preventable bloodstream infections beyond NHSN-reportable CLABSIs.

We observed that NHSN-reportable CLABSIs were not associated with preventability. This is likely a definitional issue because contaminants that are preventable are not part of the NHSN-CLABSI criteria, whereas some NHSN-CLABSI events were classified as unidentified source per clinician review and thus were not considered preventable. Neutropenia was associated with decreased likelihood of a preventable HOB episode in multivariable analysis because neutropenic patients are at increased risk for bloodstream infection due to intestinal translocation.^11^ HOB episodes that occurred after hospital day 6 were associated with increased likelihood of preventability. This could be attributed to increased risk of HAI with longer hospital stay; thus, infection prevention measures have a critical role in preventing these HOB episodes.^12^ Other factors associated with preventable HOB events in multivariable analysis included sepsis as an indication for obtaining blood culture, which had lower likelihood of preventability, whereas having urinary catheter was associated with higher likelihood of preventability. This potentially indicates that identifying factors (eg, removing Foley catheter) and intervening before progression to sepsis are critical in preventing HOB.

Our results indicate that microbiology laboratory–based HOB surveillance would be more resource efficient than CLABSI surveillance or HOB surveillance in determining the infection source and preventability. However, in the 2 study hospitals, such surveillance cannot be performed because the date of admission is not included in the laboratory database. This could be resolved by adding date of admission to the blood-culture requisition form. Implementing HOB surveillance involving assessment of source and preventability is not feasible because it requires more resources than NHSN-CLABSI surveillance.

The strengths of the study include capturing all blood cultures performed in a 6-month period and detailed chart review of almost 75% of all HOB events that occurred in the 2 hospitals. However, this study had several limitations. First, retrospective review of HOB cases limited the determination of source and preventability due to poor documentation in the medical charts. Second, this study occurred during the COVID-19 pandemic, and a significant number of patients were hospitalized with COVID-19 during the study period. Therefore, caution must be taken in generalizing the study findings to times with lower COVID-19 incidence. Similarly, the study was conducted in 2 private hospitals with inherent differences; therefore, caution should be taken in generalizing these findings to public and other private hospitals in India and other LMICs. Third, we included HOB events attributed to skin contaminants, which are not true bloodstream infections. Fourth, despite a 4:1 bed ratio, we reviewed 200 HOB cases in hospital A and 100 HOB cases in hospital B due to budget limitations. Finally, some HOB cases could have been missed due to the practice of not obtaining blood cultures prior to initiating antibiotics, which occurs commonly in resource-limited settings,^13^ and this could be a potential reason for lower HOB preventability rate observed in India compared to the US pilot study.

In conclusion, our findings suggest that HOB and NHSN-reportable CLABSI events identify the same organisms causing HAIs but that NHSN-reportable CLABSIs constitute only a minor portion of HOB events. Moreover, HOB captures preventable bloodstream infections beyond a central line as the source of HOB and thus may have utility as an HAI metric in LMIC settings. Future studies in LMICs should examine the feasibility and utility of microbiology laboratory-based HOB surveillance.

## Supplementary material

For supplementary material accompanying this paper visit https://doi.org/10.1017/ice.2023.170. click here to view supplementary material
