# Supplementary material for: Epidemiology and preventability of hospital-onset bacteremia and fungemia in 2 hospitals in India
Source: Infect Control Hosp Epidemiol. 2023 Aug 18;45(2):157–66. doi: 10.1017/ice.2023.170 (PMC10877540; doi:10.1017/ice.2023.170)
Supplement: Supplementary file 1 [file S0899823X23001708sup001.docx]

**Supplementary material**

**Supplementary Table 1: List of medical conditions specific to low and middle-income countries added to the HOB preventability framework**

| **Acute Medical Condition** | **Intrinsic Risk for Bacteremia** | **Comments** |
| --- | --- | --- |
| Acute Rhematic fever | Low |  |
| Amoebic liver abscess | Low |  |
| Arboviral infections (dengue, chikungunya, JE, Zika, Yellow fever) | Low |  |
| Bronchiolitis (children) | Low |  |
| Complicated Neurocysticercosis | Low | Consider Medium if neurological issues leading to aspiration risk, requiring foley, percutaneous endoscopic gastrostomy, etc. |
| COVID-19 | Low |  |
| Cystic Liver Lesions (echinococcus) | Low | Medium if undergoes complicate surgery |
| Dengue complicated by hemorrhagic shock | Medium-High |  |
| Enteric Fever | Low |  |
| Food poisoning/Acute Gastroenteritis illness | Low |  |
| Ingestion of Pesticides (suicide attempt) | Low-Medium | Depending on the nature and amount of pesticide/chemical |
| Leishmaniasis | Low |  |
| Malaria | Low |  |
| Myocarditis/Pericarditis | Low |  |
| Neonatal Pustulosis | Medium |  |
| Rickettssial infections (scrub typhus, murine typhus, spotted fevers) | Low |  |
| Severe malnutrition | High |  |
| Snake bites | Low |  |
| Spirochaete infections (leptospirosis, tick and louse borne relapsing fevers) | Low |  |
| Tuberculosis, Leprosy | Low |  |
| Vaccine preventable diseases (Diphtheria, Mumps, Measles, Chickenpox) | Low |  |
|  |  |  |
| **Chronic Comorbid Condition** | **Intrinsic Risk for Bacteremia** | **Comments** |
| Birthweight >= 1500g | Low |  |
| Birthweight less than 1500g | High |  |
| Lymphedema | Low |  |

**Supplementary Table 2:** **Subject Matter Experts who participated in the evaluation of the HOB preventability framework**

| **Name of the expert** | **Specialty** | **Institution, Country** |
| --- | --- | --- |
| Carry-Ann Burnham | Clinical Microbiology | Washington University in St. Louis, USA |
| Daniel Diekema | Infectious Diseases & Microbiology | University of Iowa College of Medicine, USA |
| Sumanth Gandra | Infectious Diseases | Washington University in St. Louis, USA |
| Chanu Rhee | Critical Care, Infectious Diseases | Brigham and Women’s Hospital, USA |
| Victor Rosenthal | Infectious Diseases | International Nosocomial Infection Control Consortium, Argentina |
| Lisa Saiman | Pediatric Infectious Diseases | Columbia University, USA |
| Sharmila Sengupta | Clinical microbiology | Medanta Hospital, India |
| Rachel Smith | Internal Medicine | CDC, USA |
| Cristina Vazquez Guillamet | Infectious Diseases | Washington University in St. Louis, USA |
| Mei Zheng | Infectious Diseases | Fudan University, China |

**Supplementary Box 1**

A pharmacist (FS) at Hospital A and two infection prevention nurses (PS & TS) at Hospital B were assigned to extract data from the medical charts and one of the physicians (Infectious Disease physicians [MM & PD] or Critical Care physician [MC & ZM]) at the two study hospitals reviewed the data to determine the source using clinical judgement, and rated preventability using the framework described above. After completion of the online training, pilot data collection using five HOB cases was performed by the study team at each hospital. The source and preventability assessment on the pilot cases was reviewed by the US investigators and necessary input was given prior to actual data collection. Additionally, all 300 HOB cases were reviewed by an Infectious Disease physician (SG) at Washington University School of Medicine and clarifications were requested as needed. There was reclassification for some HOB cases on SG review. For hospital A, additional information was requested for 42 HOB cases and reclassification of source/preventability occurred in 17 HOB cases. For hospital B, additional information was requested for 25 HOB cases and reclassification of source/preventability occurred in 12 HOB cases.

**Appendix 1**

**Qualitative Interview Questionnaire- EPHOBI study**

**Questions to Research staff or infection control nurse (ICN)**

1. Were there any barriers collecting blood culture data?
2. Were there any barriers collecting data on patient-days and central line-days?

**Questions to Microbiologist:**

1. Whether laboratory information system is available for obtaining blood cultures data electronically?
2. If Yes, to above question- Whether date of admission is included in addition to date of specimen collection?
3. If date of admission is not included, is it possible to incorporate into laboratory information system for each blood culture?

**Question to Microbiologist and Information Technology folks:**

1. If laboratory information system is not available, what resources will be needed and what will be the cost for the resources in Indian rupees?
2. If date of admission is not included as part of the electronic blood culture data, what resources will be needed to include that information and what will the cost for the resources in Indian rupees?
3. If date of admission is included, what resources will be needed to identify HOBs and the proportion of HOBs among total positives?

**Questions to Research staff (ICNs and clinicians)**

1. Were there any barriers collecting data to determine the source and preventability of HOB? Any potential solutions? **(for ICNs)**
2. What were the barriers to determine the source and preventability of HOB? Any potential solutions? **(for ICNs and clinicians)**
3. What was the average time needed to collect information to determine the source and preventability of one HOB? **(for ICNs)**
4. What was the average time needed to determine the source and preventability of HOB? **(for clinicians)**

**Questions to Head of Infection Control (local Study PIs)**

1. Are there potential solutions for barriers/factors affecting implementation of HOB surveillance as perceived by the research study staff (ICNs, clinicians, microbiologist and information technologist)?
2. Considering all factors what type of HOB surveillance (simple laboratory based or focused or comprehensive) is realistic to implement in your organization?
